# Varicella-zoster virus infections – antiviral therapy and diagnosis

**DOI:** 10.3205/id000019

**Published:** 2016-02-17

**Authors:** Andreas Sauerbrei

**Affiliations:** 1 Institute of Virology and Antiviral Therapy, German Consulting Laboratory for HSV and VZV, Jena University Hospital, Friedrich-Schiller University, Jena, Germany

**Keywords:** varicella-zoster virus, varicella, zoster, antiviral therapy, laboratory diagnosis

## Abstract

Varicella-zoster virus is an important human pathogen that causes varicella after primary infection and zoster after recurrence. Following primary infection, the virus remains latently for life in dorsal root and cranial nerve ganglia. Varicella and zoster are worldwide widespread diseases and may be associated with significant complications. This manuscript presents a short overview about the fundamental knowledge including the most important clinical signs, the capabilities for antiviral treatment and the spectrum of methods for laboratory diagnosis.

## 1 Fundamental knowledge

### 1.1 Virus, epidemiology and pathogenesis

Varicella-zoster virus (VZV, human herpesvirus 3 – HHV-3) is a member of the genus *Varicellovirus* within the subfamily *Alphaherpesvirinae* and the family *Herpesviridae*. It is an enveloped DNA virus with low environmental resistance and has a size of 150–200 nm [1]. The viral genome consists of double-stranded DNA with a length of 125 kb und comprises 73 open reading frames. The icosahedral capsid has 162 capsomers and is surrounded by a lipid envelope comprising of host cell components and virus-encoded glycoproteins. The virus binds via glycoproteins to cellular receptors like the mannose-6-phosphate and penetrates the cellular membrane thereafter. As with all herpesviruses, the viral replication is a complex cascade-adjusted process with sequential expression of α, β and γ genes. Viral replication takes place mainly in the nucleus of infected cells. Varicella-zoster virus has only one serotype. Despite a pronounced genetic homogeneity, there are nucleotide polymorphisms within the VZV genome leading to the classification of 5 major clades after whole genome sequencing, showing different geographical distributions [2]. The VZV DNA has numerous sequence homologies with the herpes simplex virus (HSV) genome.

Varicella-zoster virus is distributed worldwide in humans. The virus is highly contagious and is transmitted predominantly by airborne droplet infection. Infected individuals excrete the virus via saliva or conjunctival fluid from two days before the onset of varicella exanthema. The fluid of skin vesicles is also highly infectious before the lesions are completely encrusted. In case of zoster, the risk to spread the infection is significantly lower since in most cases only the vesicle fluid is infectious. Exclusively in zoster patients with particularly pronounced immunodeficiency, VZV can be shed via the pharynx. While in countries with temperate climate the majority of children develop varicella before the age of 10 years, a relatively small portion of children in tropical and subtropical areas have been demonstrated to be VZV-seropositive, and varicella affects mainly adolescents and adults [3]. Before the implementation of universal varicella vaccination in Germany in 2004, VZV seroprevalence showed a rapid increase during the first decade of life and reached between 80% and more than 90% [4]. Among adults who are more than 40 years old, only isolated individuals were susceptible to VZV. In women of child-bearing age, VZV seroprevalence has been calculated as approximately 95–97%. Risk groups for life-threatening primary VZV infections are seronegative adults, young infants from seronegative mothers, patients with immunodeficiency, unborn children in case of maternal varicella during the first 20 (24) weeks of gestation and newborns from mothers with varicella infections shortly before or after delivery. The risk of zoster is increased in elderly people, immunodeficient patients and children after varicella during pregnancy or the first year of life. 

Varicella-zoster virus is cytopathogenic during productive infection. However, after primary infection, it can establish latency in ganglion cells. Following centripedal axonal transport, circular viral DNA persists in neurons of dorsal root and cranial nerve ganglia, where it can remain quiescent for years or even decades, respectively. From there, viruses may be reactivated and may cause recurrent infections called zoster after centrifugal transport via nerve axons. The cumulative incidence of VZV reactivations leading to zoster increases significantly in older people [5] in accordance with the waning VZV-specific cell-mediated immunity in the elderly [6]. During latency, immediate early as well as early viral proteins from several open reading frames can be detected in human neurons [7]. Currently, it is assumed that there is a continuous but low level viral replication under immunologic control during VZV latency [8].

During primary infection (incubation period 10–21 days), VZV invades the body through the mucous membranes of the upper respiratory tract and undergoes the first replication in the regional lymph nodes followed by a primary lymphocyte-associated viremia 4–6 days *post infectionem* (p.i.) and a peripheral blood mononuclear cell-associated secondary viremia 10–14 days p.i. disseminating the virus to the skin [9]. 

### 1.2 Clinical signs

Nearly all cases of the primary VZV infection result in varicella [10]. In temperate climates, the disease peaks during winter and early spring. Before the universal varicella vaccination was introduced, approximately 750,000 varicella cases per year were observed in Germany [11]. The clinical pictures range from harmless varicella during childhood to severe courses in immunodeficient patients of all age groups. The disease begins suddenly with an itchy rash and partially with moderate fever. Varicella exanthema is characterized initially by pinhead to pea sized erythematous macules developing consecutively to papules, water-clear vesicles, yellowish pustules and crusts. There are always different stages of exanthema simultaneously resulting in the picture of a “starry sky”. As a rule, the contagiosity of varicella ends approximately 5–7 days after onset of exanthema with complete crusting of skin vesicles. After about 2 weeks, the exanthema is completely healed. Varicella complications have rarely been observed in immunocompetent pre-school children [12]. However, the disease is a special risk for patients with impaired cellular immune function, e.g. patients with oncological diseases, organ or bone marrow transplantation, autoimmunopathies, congenital immune defects or individuals infected with the human immunodeficiency virus [13], [14]. The most common complications are related to secondary bacterial infections, neurological and hematological manifestations. In addition, varicella during pregnancy is associated with high risk of maternal pneumonia and congenital transmission of the virus leading to severe fetal sequelae [15]. After varicella infection between 5 and 20 (24) gestational weeks, a congenital varicella syndrome with 30% mortality can be expected in 1–2% of the cases with the main clinical symptoms of segmental cicatricial skin lesions, neurological diseases, eye diseases and limb hypoplasia. In case of maternal varicella between 5 days before and 2 days after delivery, there is the high risk of neonatal varicella with fatal outcome in up to 20% of the cases without antiviral therapeutic intervention. The repeated occurrence of varicella, so-called secondary varicella, is almost exclusively observed in patients with impaired cellular immune response. Breakthrough diseases can be considered as a new manifestation of varicella caused by the wild-type virus and occurring at the earliest at 42 days after single varicella vaccination with a prevalence of 4(–9)% in persons vaccinated annually [12]. Most breakthrough diseases are very mild and the infectivity is low [16]. 

Herpes zoster, also referred shortly as zoster, always reflects a recurrent VZV infection after endogenous virus reactivation. In Germany, zoster prevalent with more than 400,000 cases per year is one of the most common viral skin infections [17]. The study group for varicella at the Robert Koch-Institute (RKI), Berlin (Germany), has reported an increasing incidence of zoster especially in people aged over 50 years during the last several years, but this trend began before the universal varicella vaccination has been recommended [18]. Zoster is preceded by a prodromal phase with burning pains and/or sensory disturbances in the area of one to three adjoining dermatomes. The disease begins with skin erythema followed by characteristic grouped papules developing to vesicles arising during an interval of 1–5 days. Afterwards, the vesicles dry out over 7–12 days. In immunodeficient patients, the disease can follow a chronic course accompanied by skin lesions persisting for months and occurring repeatedly. Zoster is predominantly localized in thoracic skin regions, but with increasing age the innervation areas of trigeminal nerve are affected. Zoster disease is more severe and more frequently associated with complications in immunocompromised patients. Important complications are neurological manifestations, hemorrhagic and necrotic skin changes, bacterial super-infections, disseminations of infection, and inclusion of eyes or ears [19]. Pains lasting longer than 4 weeks and occurring again after a pain-free interval are designated as postzosteric neuralgia caused by an irreversible necrosis of ganglion cells. A peripheral sensitization of nociceptive c fibers followed by central sensitization of spinal nociceptive neurons and a degeneration of nociceptive c fibers as result of inflammation are discussed as pathomechanisms [11]. Zoster during pregnancy is generally not hazardous for the infant.

## 2 Antiviral therapy

### 2.1 Antiviral agents in clinical use

Replication of VZV in infected cells can be blocked effectively by the administration of antiviral agents. An early administration especially in zoster may reduce the damage of tissue, and thus the destruction of affected ganglion cells can be diminished or even prevented. Primarily, the acyclic nucleoside analogs acyclovir including its prodrug valaciclovir, famciclovir (prodrug of penciclovir) and the cyclic nucleoside analog brivudin ((E)-5-(2-bromovinyl)-2'-deoxyuridine, BVDU) are available for antiviral therapy of VZV infections (Table 1 (Tab. 1)). The specificity of antiviral activity is based on the phosphorylation of these inhibitors by the viral thymidine kinase (TK) to their mono- (acyclovir, penciclovir) or diphosphates (brivudin), while the further phosphorylation steps to the triphosphate are catalyzed by cellular enzymes. Thus, the spectrum of activity is defined by the presence of the key enzyme, the viral TK. The triphosphates of the nucleoside analogs inhibit and fix the viral DNA polymerases (pol) and are incorporated as “false” substrate into the growing DNA chain. In case of acyclovir/valaciclovir, this results in chain termination due to the absence of the hydroxy group in 3’ position essential for further linking. In other nucleoside analogous compounds, their incorporation into DNA is possible.

#### Acyclovir

 It is the standard therapeutic agent for antiviral treatment of VZV infections, but the oral bioavailability is only 15–30%. Oral treatment is recommended for varicella in risk patients and zoster disease in immunocompetent patients. In severe VZV infections, especially in immunodeficient patients, acyclovir has to be administered intravenously (i.v.). After i.v. administration, side effects on the central nervous system (CNS) have been observed occasionally, whereas the oral medication can be associated with gastrointestinal symptoms. Substances with kidney toxicity should not be combined simultaneously with acyclovir. Laboratory kidney and liver parameters have to be monitored.

#### Valaciclovir

The prodrug (l-valyl ester) of acyclovir is administered orally and is converted into acyclovir by the hepatic valaciclovir hydrolase. Valaciclovir has an oral bioavailability of 54% resulting in three to four times higher drug concentrations than oral acyclovir. The consequences are longer dosing intervals and a better compliance. The administration of valaciclovir is approved for antiviral treatment of zoster in immunocompetent adults, but the drug is not approved for the use during childhood and adolescence. Possible side effects are similar to those after medication of acyclovir.

#### Famciclovir

This inactive diacethyl ester prodrug of penciclovir arises by separation of two ester groups in small intestine and liver. Penciclovir is an acyclic nucleoside analog (exchange of the ether oxygen atom in the acyclic side chain by a methyl bridge) derived from gancyclovir. Since the oral absorption is very low, penciclovir is only used for topic antiviral treatment of local HSV infections. In contrast, famciclovir has a bioavailability of 77% after oral administration. It is used for antiviral therapy of zoster in immunocompetent adults and immunosuppressed patients from the age of 25 years. Similar to valaciclovir, famciclovir is not approved in childhood and adolescence. In rare cases, taking of famciclovir can lead to headaches, mental confusion and nausea.

#### Brivudin ((E)-5-(2-bromovinyl)-2'-deoxyuridine)

This cyclic nucleoside analog, converted to its mono- and diphosphate by the viral TK, is administered orally and has a bioavailability of approximately 40%. It is used for the antiviral treatment of zoster in immunocompetent adults. Since the safety profile is unknown, brivudin is not approved for antiviral therapy in children and adolescents. Therefore, the risk-benefit ratio should be examined carefully before the agent can be used in children and adolescents, and the parents have to be informed (off-label use). Brivudin is generally well tolerated, but gastrointestinal disturbances, impairment of renal function, increasing of liver enzymes and reversible changes of blood cell count may occur. A simultaneous administration of 5-fluorouracil or other 5-fluoropyrimidines results in an enhanced and possibly dangerous toxicity. 

#### Foscarnet (trisodium phosphonoformate)

The pyrophosphate analog foscarnet inhibits the viral DNA pol of numerous DNA and RNA viruses by the prevention of the pyrophosphate exchange. Since this drug is not required to be metabolized for its antiviral activity, it has also an effectiveness against TK-negative VZV strains resistant to nucleoside analogs. That is why foscarnet is recommended for alternative antiviral treatment in suspected clinical resistance to acyclovir expecting rarely in immunosuppressed patients with severe zoster courses. Disturbances of renal function and toxic caused ulcers of urogenital mucosa have to be considered as important side effects.

### 2.2 Development of resistance

Resistance of VZV against antiviral drugs as acyclovir or foscarnet has been observed only in immunodeficient patients suffering from zoster e.g. in the acquired immune deficiency syndrome or under immunosuppression due to cancer diseases or transplantation [20], [21], [22]. For the development of resistance, the impaired immune response and long-term administration of drugs in the context of antiviral treatment or chemoprophylaxis are of crucial significance. The weakened immune response leads to a longer virus replication with the spontaneous development of an increasing number of resistant virus mutants followed by selection of resistant viruses that cannot be eliminated by the impaired immune response [23]. Generally, resistances are associated with non-synonymous mutations localized within the gene of the respective target molecule or within the gene of proteins responsible for the metabolisation or the effectiveness of antiviral agents. For acyclovir and the related nucleoside analogs, resistance is based almost exclusively on non-synonymous mutations of TK gene (*UL36*) and rarely, mostly in connection with resistance against foscarnet, on amino acid (aa) changes in the DNA pol gene (*UL28*) [24]. In contrast to the TK that is not required for the replication of VZV, the DNA pol is essential for the viral replication cycle. According to previous findings, VZV strains resistant to acyclovir due to mutations in the TK gene are always cross-resistant to brivudin [22]. Until now, there are only a few studies in the literature in which the significance of TK and DNA pol mutations was verified by phenotypic findings. The main reason is that VZV can only be isolated rarely in cell culture from patient samples, at best, from vesicle fluid. 

#### Thymidine kinase gene

The thymidine kinase gene of VZV has a size of 1,026 bp and is coding for 342 aa. This gene contains two conserved regions, one nucleotide (ATP)-binding site (aa 12–29) and one nucleoside (substrate)-binding site (aa 129–145). Contrary to TK of HSV-1 and HSV-2, only a low number of natural polymorphisms have been identified in the VZV TK, but it is important to differentiate each of them from resistance mutations [22]. Compared to the VZV strain Dumas (GenBank accession No. X04370.1, clade 1) frequently used as reference, all European wild-type strains comprise the aa polymorphism S288L. Comparable with HSV [25], the resistance mutations of VZV TK gene are assigned to three phenotypes (i) TK negative (TK-, no TK activity detectable, occur most frequently), (ii) TK reduced (TKr, diminished TK activity, 1–15% of normal activity) and (iii) TK altered (TKa, altered TK substrate specificity, no phosphorylation of acyclovir and other nucleoside analogs). Resistance to acyclovir may be caused by stop codons, frameshifts (deletions or insertions) or aa substitutions inside and outside of conserved gene regions. Since only a few validated resistance mutations have been reported in the literature to now, novel or unknown mutations must always be expected when clinically or phenotypically resistant VZV strains are analyzed genotypically. 

#### DNA polymerase gene

This gene with a size of 3,585 bp is coding for 1,195 aa. There are eight conserved regions designed to I to VII and A. Similar to the TK gene, only a small number of natural polymorphisms have been reported for DNA pol [22]. Resistance-related aa substitutions are localized mostly in conserved gene centers. So far, little research has been done on natural polymorphisms and resistance mutations of the VZV DNA pol gene.

## 3 Laboratory diagnosis

### 3.1 Detection of virus

Varicella-zoster virus-positive samples have to be considered as dangerous goods of the category B, risk group 2 to be shipped according to UN 3373 regulations. Shipment is possible at room temperature, and cooling is only recommended if samples are intended for virus isolation in cell culture [26]. Acute VZV infection is diagnosed (Table 2 (Tab. 2)) by detection of the viral DNA using polymerase chain reaction in vesicle fluids, cerebrospinal fluids, tissues, bronchoalveolar lavages, EDTA blood, amniotic fluids, or intraocular fluids (retinal necrosis) [27]. Isolation of VZV, only possible in a few cell types such as human embryonic fibroblasts, is time-consuming, requires a high degree of experience and has no clinically relevant sensitivity. In most cases, only vesicle fluids containing high virus load are suitable for viral isolation. For successful viral isolation, an early and careful taking as well as an optimal transport of samples are essential. Identification of viral isolates is carried out properly by immunofluorescence using monoclonal fluorescein-labelled antibody. Direct qualitative detection of VZV antigens by use of commercial detection systems may provide results within a few hours, but this method has a reduced sensitivity and specificity. Procedures for direct detection of VZV including nucleic acids or antigens do principally not allow any differentiation between primary and recurrent infection. Discrimination between VZV wild-type and vaccine strains can be performed by genotyping using restriction fragment length polymorphism analysis or sequencing [28], [29].

### 3.2 Detection of antibodies

Serological VZV diagnosis is especially indicated if susceptible persons have to be identified. Because of the high rates of seroconversion, the determination of antibody status is not necessary after varicella vaccination in healthy children, adolescents, and adults in contrast to immunodeficient vaccinees and healthcare workers [30]. In the daily laboratory practice, ligand assays or immunofluorescence tests are common for the determination of VZV-specific IgG antibodies (Table 2). Regardless of the test used, each result interpreted as anti-VZV IgG positive by the respective laboratory can be used as criterion of immunity against varicella and individuals with borderline findings should be classified as “not immune“. Commercially available test kits differ with respect to sensitivity. Therefore, high sensitive tests such as special glycoprotein enzyme-linked immunoassays (ELISA) or the fluorescence antibody membrane antigen test (FAMA) should be used to control immune status after varicella vaccination and for vaccine studies [31], [32]. Primary VZV infection can be diagnosed by the determination of VZV IgG seroconversion after sequential blood samples were obtained. Anti-VZV IgM will be detectable, usually in combination with anti-VZV IgG, at the earliest from the fourth day after onset of disease. Even though anti-VZV IgM is commonly used in practice to confirm active VZV infection, it has to bear in mind that IgM antibodies will be detectable with significant delay after onset of varicella exanthema and only in maximally 50–60% of patients with zoster [27]. In addition, numerous commercial VZV IgM immunoassays have a reduced sensitivity and may show false-positive results caused by cross-reactions with other herpesviruses, in particular with HSV [15]. Anti-VZV IgA may be determined frequently in persons infected latently with VZV, but high titer values exclusively correlate with zoster disease. Intrathecal VZV-specific IgG antibodies may be of significance for retrospective diagnosis of VZV-associated CNS infections [33]. Determination of VZV IgG avidity allows the differentiation between primary (varicella) and recurrent infections (zoster), but there is only limited experience to this [34].

### 3.3 Resistance testing

In case of VZV infections, an antiviral treatment failure due to resistance is assumed if there are no clinical improvements detectable during the administration of the antiviral medication, mostly acyclovir within 10 (–21) days [21], [24], [35]. In these cases, an alternative treatment with foscarnet is indicated. In parallel, genotypic resistant testing and, if a viral isolate can be established in cell culture, phenotypic resistant testing should be carried out. Because of the high expenditure of time (at least 3 weeks), the phenotypic resistance testing has mostly no clinical relevance, but the procedure can help to characterize novel mutations or gene variations which are so far unclear with respect to their significance for any resistance. 

#### Phenotyping

Phenotyping has been considered as gold standard for resistance testing of VZV, but the VZV isolation in cell culture has low sensitivity. The plaque reduction assay has been established as the method of choice [22]. After adding the antiviral compound to be tested in descending dilution series, the 50% effective concentration (EC_50_) is estimated inducing a 50% inhibition of viral replication. For the evaluation of possible resistance, a susceptible VZV reference strain has to be tested in each experiment as control. It is a crucial advantage that phenotyping allows an unambiguous interpretation of the results. However, the procedures used are time- and material-consuming as well as non-standardized. In practice, phenotypic resistance testing can only be realized if swabs can be obtained from vesicle fluids from which the virus can be isolated in cell culture. For interpretation of the results, the most common and reliable procedure for nucleoside analogs is to classify VZV strains as resistant if the mean EC_50_ measured is three to five times higher than the corresponding value of sensitive control strain [36]. For resistance to foscarnet, EC_50_ values >300 µM have been proven to be sound [37]. 

#### Genotyping

By analogy with HSV, genotyping resistance testing of VZV is performed by means of amplification and sequencing TK and DNA pol genes [25], [38]. For the identification of non-synonymous mutations, sequence data must be compared with the published sequences of a susceptible reference strain available in the Genbank (e.g. VZV strain Dumas, accession No. X04370.1). The advantage of genotyping is a considerably shorter delay (approximately 2 days) in comparison to phenotyping and the direct testing of patient samples without virus isolation in cell culture. The restricted quantity of viral DNA may have a limiting effect. A great disadvantage is the fact that there is only little information available about assured resistance-associated aa substitutions. That is why only stop codons or frameshift mutations can be interpreted without doubt as related to resistance. In addition, the analysis of genotypic resistance may be difficult when a mixture of viral strains with different genotypes is present. Therefore, it is a problem in clinical practice to define a questionable resistance of VZV strains on the basis of genotyping results alone. Recently, it has become apparent that phenotypic testing of recombinant VZV isolates is the best method to validate the significance of mutations for any resistance [39].

## Notes

### Conflict of interest

The author declares that he has no competing interests.

### Acknowledgement

This review was supported by the scientific advisory board for Antiviral Therapy of the German Association for the Control of Virus Diseases and the Society of Virology.

## Figures and Tables

**Table 1:**
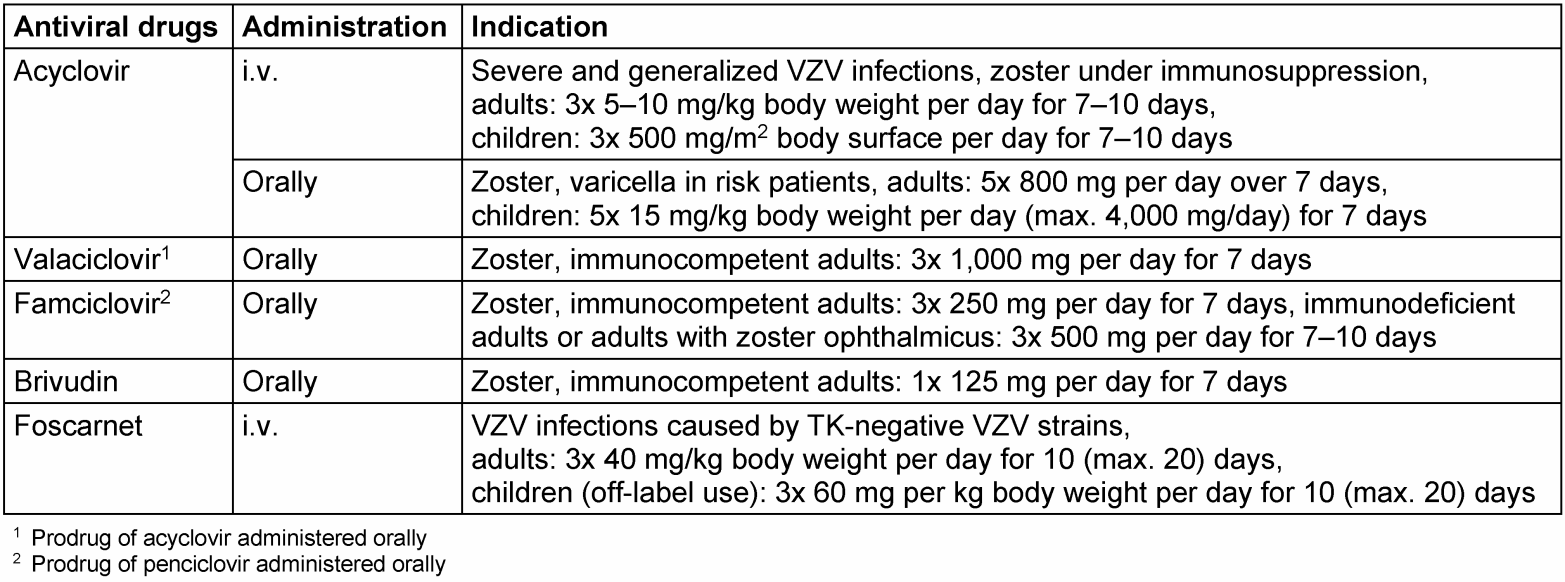
Antiviral drugs against VZV in clinical use

**Table 2:**
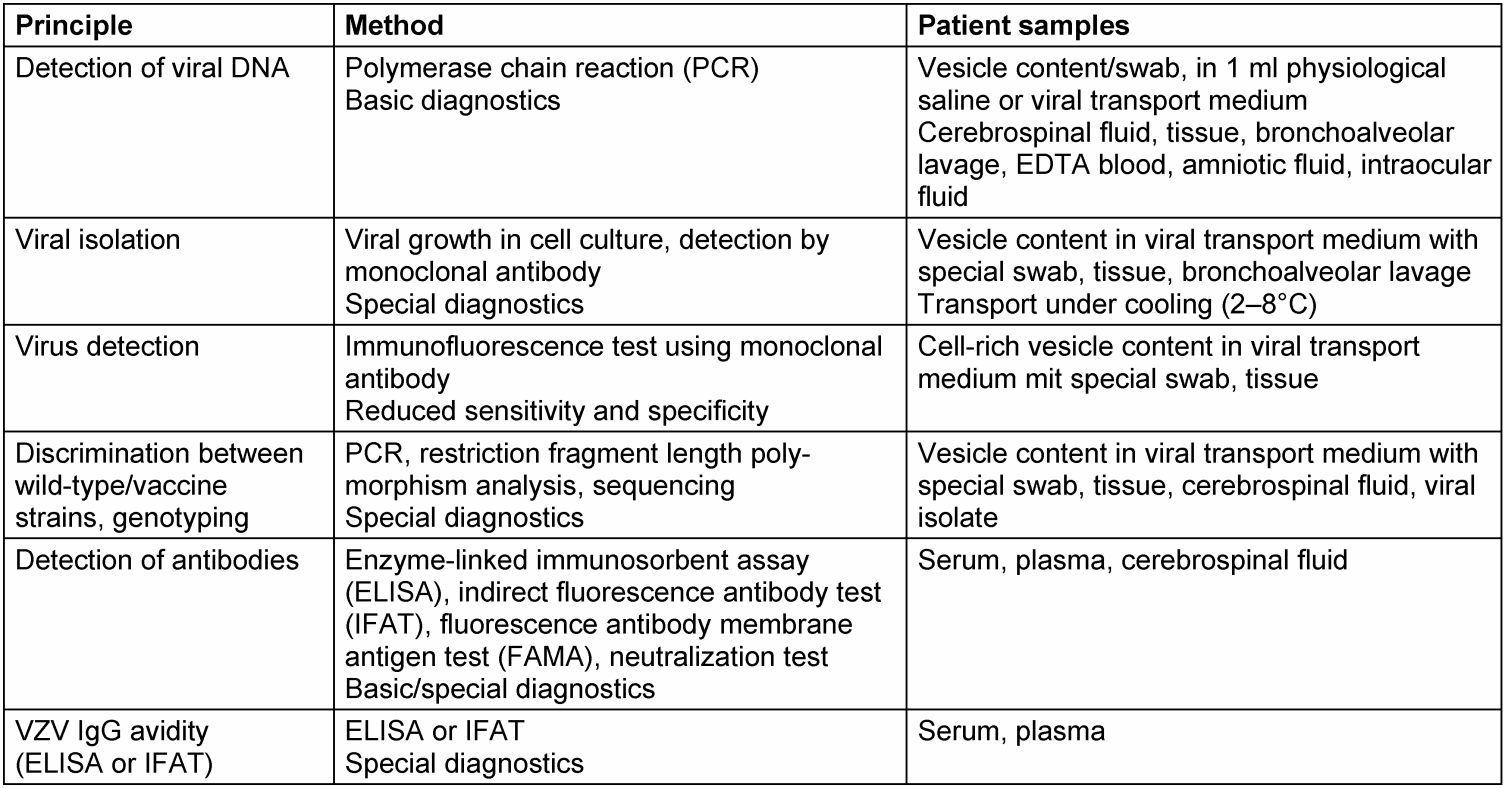
Methods for laboratory diagnosis of VZV infections
